# Acute postoperative pain management after cardiothoracic surgery: a bibliometric analysis and future directions

**DOI:** 10.1186/s13019-025-03734-x

**Published:** 2025-11-27

**Authors:** Jingyun Wang, Youliang Deng, Qin Chen, Feng Chen, Hong Li

**Affiliations:** Department of Anesthesiology, Second Affiliated Hospital of Army Medical University, Chongqing, 400037 China

**Keywords:** Cardiothoracic surgery, Regional nerve blocks, Acute postoperative pain, Bibliometric analysis

## Abstract

**Background:**

Acute postoperative pain following cardiothoracic surgery remains a significant clinical challenge with numerous studies on its management published over the past decades. This study aims to summarize research progress and identify future research trends in acute postoperative pain management after cardiothoracic surgery using retrospective bibliometric analysis.

**Methods:**

Relevant studies on acute postoperative pain management following cardiothoracic surgery were retrieved from the Science Citation Index Expanded of the Web of Science Core Collection database, covering the period from January 1, 2004, to January 1, 2024. VOSviewer was used to perform and visualize analyses of total publications, contributing countries, research institutions, journals, authors, co-citation networks, and keyword co-occurrence. CiteSpace was employed to visualize burst analyses of co-cited references and keywords.

**Results:**

From 2004 to 2024, a total of 740 papers on acute postoperative pain management in cardiothoracic surgery were published, authored by 4,054 researchers from 911 organizations across 50 countries. These papers appeared in 196 journals and cited 10,902 references from 2,242 journals. The most high-yield publication year, countries, institutions, journals, authors were 2021 (n=96), China (n=173), Cleveland Clinic (n=14), *Journal of Cardiothoracic and Vascular Anesthesia*(n=108), and Chauhan, S (n=6), respectively. Regional nerve blocks were one of the significant research topics in acute postoperative pain management following cardiothoracic surgery. In recent years, the keywords with the strongest citation burst were *erector spinae plane block, enhanced recovery, thoracic paravertebral block, nerve block, plane block, and liposomal bupivacaine*.

**Conclusion:**

This study provides a bibliometric overview of the development of acute postoperative pain management in cardiothoracic surgery over the past two decades. Overall, research in this field has shown a steady increase globally. The future research hotspots might be *erector spinae plane block*, *enhanced recovery*, *thoracic paravertebral block*, *nerve block*, and *liposomal bupivacaine*.

**Supplementary Information:**

The online version contains supplementary material available at 10.1186/s13019-025-03734-x.

## Background

Cardiothoracic surgery is crucial for treating various diseases, including lung cancer, esophageal cancer, mediastinal tumors, chest trauma, and rheumatic heart disease. However, managing severe acute pain after these procedures remains a significant challenge [1–3]. The incidence of moderate-to-severe pain after thoracic surgery has been reported to reach as high as 85% [4]. Despite advancements in surgical techniques, such as the increasing adoption of minimally invasive procedures (e.g., video-assisted thoracoscopic surgery and robotic-assisted surgery), acute postoperative pain remains a significant issue [5, 6]. Poorly controlled acute postoperative pain not only affects patient’s recovery, but also prolongs hospitalization and increases the healthcare costs [7, 8]. In contrast, effective postoperative analgesia can mitigate stress responses, reduce postoperative complications, and improve patient prognosis [9, 10]. As a result, research on acute postoperative pain management in cardiothoracic surgery has grown steadily over the past two decades. A variety of analgesic strategies are available for acute postoperative pain management in cardiothoracic surgery, encompassing multiple drug classes and routes of administration [11–14]. We aim to provide an overview of the progress in this field.

Bibliometrics is the study of academic publishing that uses statistics to describe publishing trends and to highlight relationships between the published works [15]. In the medical field, bibliometric studies are used to understand the trajectory of diseases, the associated research hotspots, and potential future research directions [16, 17]. More importantly, bibliometrics can provide valid evidence for making clinical decisions, determining future trends, and guiding future research directions in anesthesia and pain management. Over the years, bibliometric research findings have been reported, such as postoperative pain management of cesarean section [18] and the dynamics of acute postsurgical pain [19]. However, there is still no bibliometric study on acute postoperative pain management in cardiothoracic surgery. The present study aims to summarize the development and trends in research on acute postoperative pain management after cardiothoracic surgery.

## Methods

### Data Sources and Search Strategies

**Fig. 1:**
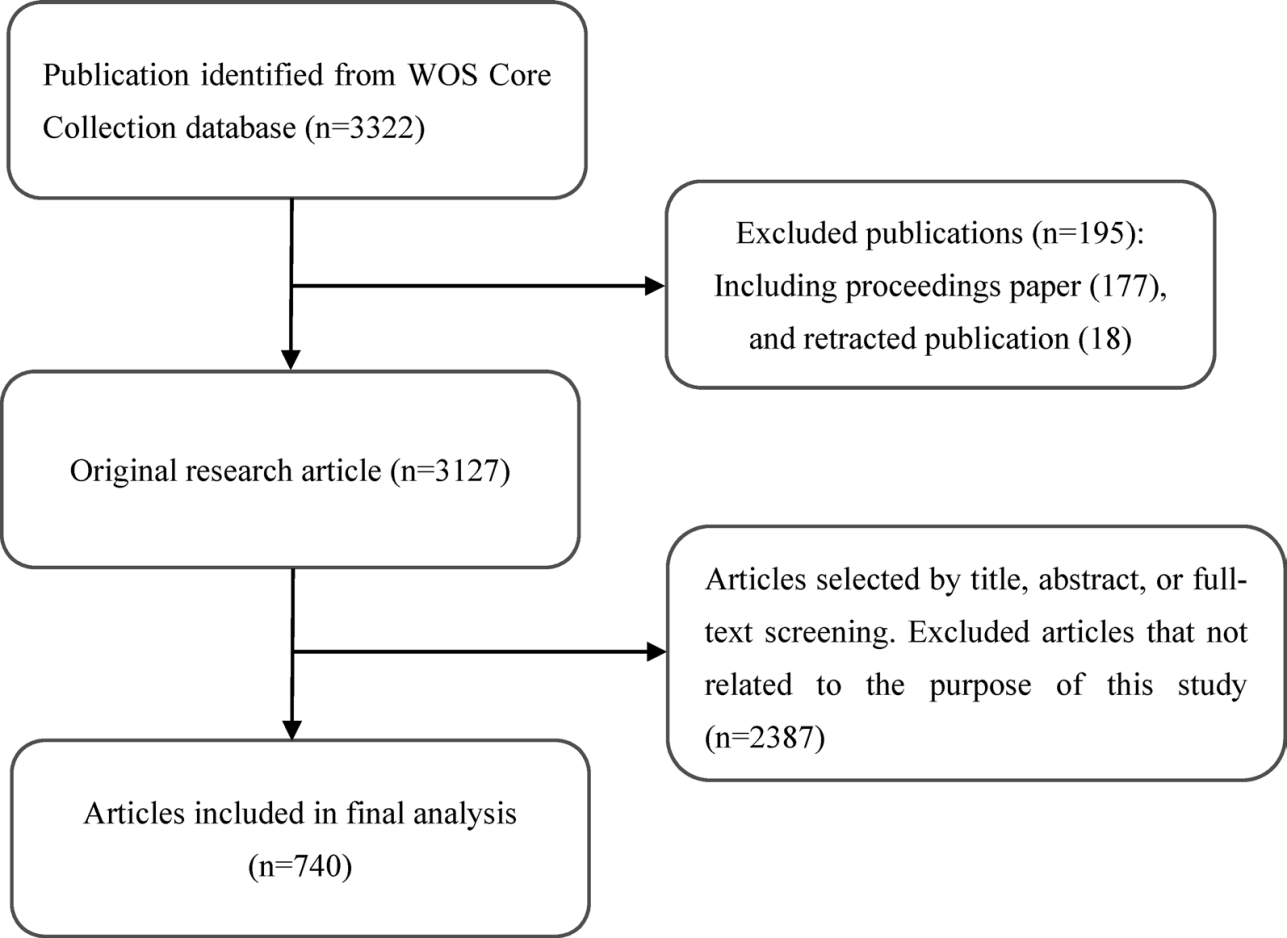
Flowchart for the publication selection included in this study

The original research articles were retrieved from the Web of Science (WOS) Core Collection database using an advanced search strategy. The time span was from January 1, 2004, to January 1, 2024, the publications type was limited to article, and the language was limited to English. In addition, the Boolean operators “AND” and “OR” were used to construct retrieval strategies. The specific retrieval strategies are detailed in Supplementary Table S1. We excluded reviews, preprints, conference abstracts, proceeding papers, editorials, letters, protocols, and articles not available in English. After the initial data search, two researchers independently screened all the manuscripts to verify their relevance. The eligible studies must concentrate on the management of acute postoperative pain in cardiothoracic surgery; studies not related to this focus will be excluded. 3322 publications met the inclusion criteria. Finally, 740 articles were further assessed. The screening process is shown in Fig. 1.

#### Bibliometric Analysis

The following bibliometric information was collected, including year of publication, country, institution, source journal, journal impact factor, authorship, total citation count, reference, and keywords. The VOSviewer software and CiteSpace software were used for bibliometric analysis. VOSviewer, developed by Van Eck and Waltman [20], is widely used for network and cluster analysis visualization. Using VOSviewer (version 1.6.20), we performed analyses of total publications, contributing countries, research institutions, journals, and authors, and we generated maps for reference co-citation analysis and keyword co-occurrence analysis. CiteSpace was developed by Chen [21] to provide two complementary visualizations: cluster view and time zone view. With CiteSpace (v. 6.4.R1), we created two analytical maps: co-cited reference bursts and keyword bursts.

## Results

### Publications by year

**Fig. 2:**
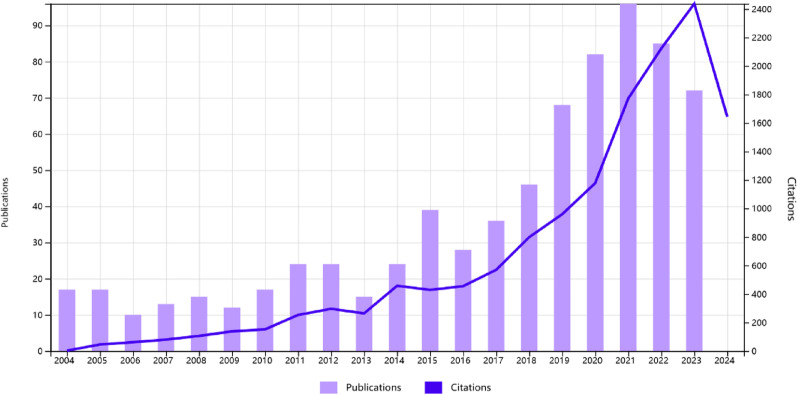
Annual publications and citations on acute postoperative pain management following cardiothoracic surgery over 20 years

The 740 papers analyzed in this study were written by 4054 authors from 911 organizations in 50 countries, and published in 196 journals. Meanwhile, the 740 papers cited 10,902 references from 2242 journals. As shown in Fig. 2, there has been a significant increase in the number of papers in this field in the last few years, and the year with the most publications (*n* = 96) was 2021. The studies published in 2021 had the highest number of total citations (*n* = 1774).

## Analysis of countries

In this study, we have conducted a bibliometric analysis of countries where the 740 papers were published. The included papers originated from 50 countries. The top 10 high-yield countries are shown in Table 1. Among them, China was the most productive country, with a total of 173 (23.4%) papers published, which received 2,370 citations. The United States was the second most productive country, with 137 papers and 2,867 citations. Although Egypt published only 25 papers, these papers have been cited a total of 670 times, with an average of 26.8 citations per paper.

**Table 1:** Top 10 high-yield countries by publications on acute postoperative pain management following cardiothoracic surgery

Rank	Country	Documents, *n* (%)	Total citations	Average citation/publication
1	China	173(23.4%)	2370	13.6
2	United States	137(18.5%)	2867	20.9
3	Turkey	79(10.6%)	1447	18.3
4	Japan	49(6.6%)	858	17.5
5	Republic of Korea	35(4.7%)	580	16.6
6	Italy	33(4.5%)	782	23.7
7	England	33(4.5%)	678	20.5
8	Canada	30(4.1%)	616	20.5
9	Egypt	25(3.4%)	670	26.8
10	Germany	21(2.8%)	355	16.9

## Analysis of institutions

Between January 2004 and January 2024, a total of 911 institutions contributed to studies on postoperative pain management following cardiothoracic surgery. Table 2 presents the top 10 institutions ranked by publication volume and citation frequency.

**Table 2:** Top 10 high-yield institutions on acute postoperative pain management following cardiothoracic surgery

Rank	Institution	Documents	citations	average citation/ publication	Country
1	Cleveland Clinic	14	412	29.4	United States
2	Mayo Clinic	12	252	18	United States
3	University of Health Sciences	12	225	18.7	Turkey
4	Nanjing Medical University	11	82	7.5	China
5	University of Montreal	9	191	21.2	Canada
6	Stanford University	9	129	14.3	United States
7	Anhui Medical University	8	171	21.3	China
8	Duke University	8	146	18.3	United States
9	University of Science and Technology of China	8	96	12	China
10	Sungkyunkwan University	7	219	31.3	South Korea

The Cleveland Clinic ranked first, with 14 publications, accumulating 422 citations and achieving an average of 29.4 citations per paper, which highlights its significant academic impact in this field. Other institutions with more than 10 publications include Mayo Clinic, University of Health Sciences, and Nanjing Medical University. The top 10 publishing institutions are distributed across five countries: the United States (4), China (3), Canada (1), Turkey (1), and South Korea (1).

## Analysis of journals

We performed a bibliometric analysis of the journals of the extracted articles. Table 3 shows the top 10 journals by publication volume and citation frequency, which mainly focus on cardiothoracic surgery, anaesthesia, and pain management. The journal with the highest number of publications was the *journal of cardiothoracic and vascular anesthesia*, which published 108 papers with 2,829 citations and an average of 26.2 citations per paper. On the other hand, the journal with the highest average citation per publication was the *Journal of Clinical Anesthesia*, which published 13 papers with an average of 32.5 citations per paper. This journal mainly focuses on all aspects of anesthesia practice and belongs to Q1 zone according to the Journal Citation Reports zoning. It has an impact factor (IF) of 5, which is significantly higher than other journals.

**Table 3:** Top 10 journals on acute postoperative pain management following cardiothoracic surgery

Rank	Journals	Paper count	Total citation	Average citation/ publication	IF of the journal In 2023
1	Journal of cardiothoracic and vascular anesthesia	108	2829	26.2	2.3
2	Journal of thoracic disease	29	397	13.7	2.1
3	Journal of pain research	27	347	12.8	2.5
4	BMC anesthesiology	25	324	13	2.3
5	Annals of thoracic surgery	21	589	28	3.6
6	Interactive cardiovascular and Thoracic surgery	17	387	22.8	1.6
7	European journal of anaesthesiology	14	412	29.4	4.2
8	European journal of cardio-Thoracic surgery	14	408	29.1	3.1
9	Journal of clinical anesthesia	13	423	32.5	5
10	Journal of cardiothoracic Surgery	13	204	15.7	1.5

### Analysis of authors

The top 10 high-yield authors with the highest number of publications on acute pain management after cardiothoracic surgery from 2004 to 2024 are listed in Table 4. Chauhan, S form All India Inst Med Sci was the most prolific author with 6 papers that received 422 citations. In terms of citations per paper, Chauhan, S also ranked first, with an average of 26.2 citations per paper. The majority of his studies focused on regional nerve blocks in postoperative analgesia in cardiac surgery [22–24]. Sessler, D.I. from UTHealth Houston ranked second, with 6 papers and a total of 123 citations.

**Table 4:** Top 10 high-yield authors on acute postoperative pain management following cardiothoracic surgery

Rank	Authors	Affiliations	Documents	Citations	Average citation/publication
1	Chauhan, S	All India Inst Med Sci	6	422	70.3
2	Sessler, D.I.	UTHealth Houston	6	123	26.2
3	Martorella, G	Florida State Univ	6	111	18.5
4	Sazak, H	Univ Hlth Sci	6	42	7
5	Feng, Y	Peking Univ	5	171	34.2
6	Zhang, Y	Nanchang Univ	5	144	28.8
7	Boitor, M	McGill Univ	5	117	23.4
8	Gélinas, C	McGill Univ	5	117	23.4
9	Alagöz, A	Univ Hlth Sci	5	35	7
10	Zengin, M	Ankara Etlik City Hosp	5	35	7

### Reference co-citation analysis and reference burst analysis

The term co-cited reference refers to two articles that are both cited by a third article, forming a co-citation connection [25]. Reference co-citation analysis is used to detect and analyse the evolution and trends within a research field. In this study, the top 10 co-cited references are shown in Table 5, and the range of the number of citations is from 39 to 88. Among these top 10 co-cited references, five are reviews and five are original articles with a focus on methods for postoperative analgesia in cardiothoracic surgery. The review by Davies et al. titled “*A comparison of the analgesic efficacy and side-effects of paravertebral vs. epidural blockade for thoracotomy–a systematic review and meta-analysis of randomized trials*”, published in 2006, is the most cited article, with 88 citations all together. The second is “*A Systematic Review of Randomized Trials Evaluating Regional Techniques for Postthoracotomy Analgesia*” authored by Joshi et al. in 2008 in the journal *Anesthesia & Analgesia*, with 87 citations.

**Table 5:** Top 10 most co-cited references on acute postoperative pain management following cardiothoracic surgery

Rank	Citations	Co-cited reference	Journal	Publication Year	type
1	88	A comparison of the analgesic efficacy and side-effects of paravertebral vs. epidural blockade for thoracotomy—a systematic review and meta-analysis of randomized trials	Br J Anaesth	2006	Review
2	87	A Systematic Review of Randomized Trials Evaluating Regional Techniques for Postthoracotomy Analgesia	Anesthesia & Analgesia	2008	Review
3	68	The Erector Spinae Plane Block: A Novel Analgesic Technique in Thoracic Neuropathic Pain	Reg Anesth Pain Med	2016	Article
4	59	Postoperative pain and quality of life after lobectomy via video-assisted thoracoscopic surgery or anterolateral thoracotomy for early stage lung cancer: a randomised controlled trial	Lancet Oncol	2016	Article
5	56	Serratus plane block: a novel ultrasound-guided thoracic wall nerve block	Anaesthesia	2013	Article
6	53	Preventing and Treating Pain after Thoracic Surgery	Anesthesiology	2006	Review
7	47	Single-injection thoracic paravertebral block for postoperative pain treatment after thoracoscopic surgery	Br J Anaesth	2005	Article
8	44	Acute pain after thoracic surgery predicts long-term post-thoracotomy pain	Clin J Pain	1996	Article
9	41	Guidelines for enhanced recovery after lung surgery: recommendations of the Enhanced Recovery After Surgery (ERAS^®^) Society and the European Society of Thoracic Surgeons (ESTS)	Eur J Cardiothorac Surg	2019	Guideline
10	39	Acute pain management for patients undergoing thoracotomy	Ann Thorac Surg	2003	Review

**Fig. 3:**
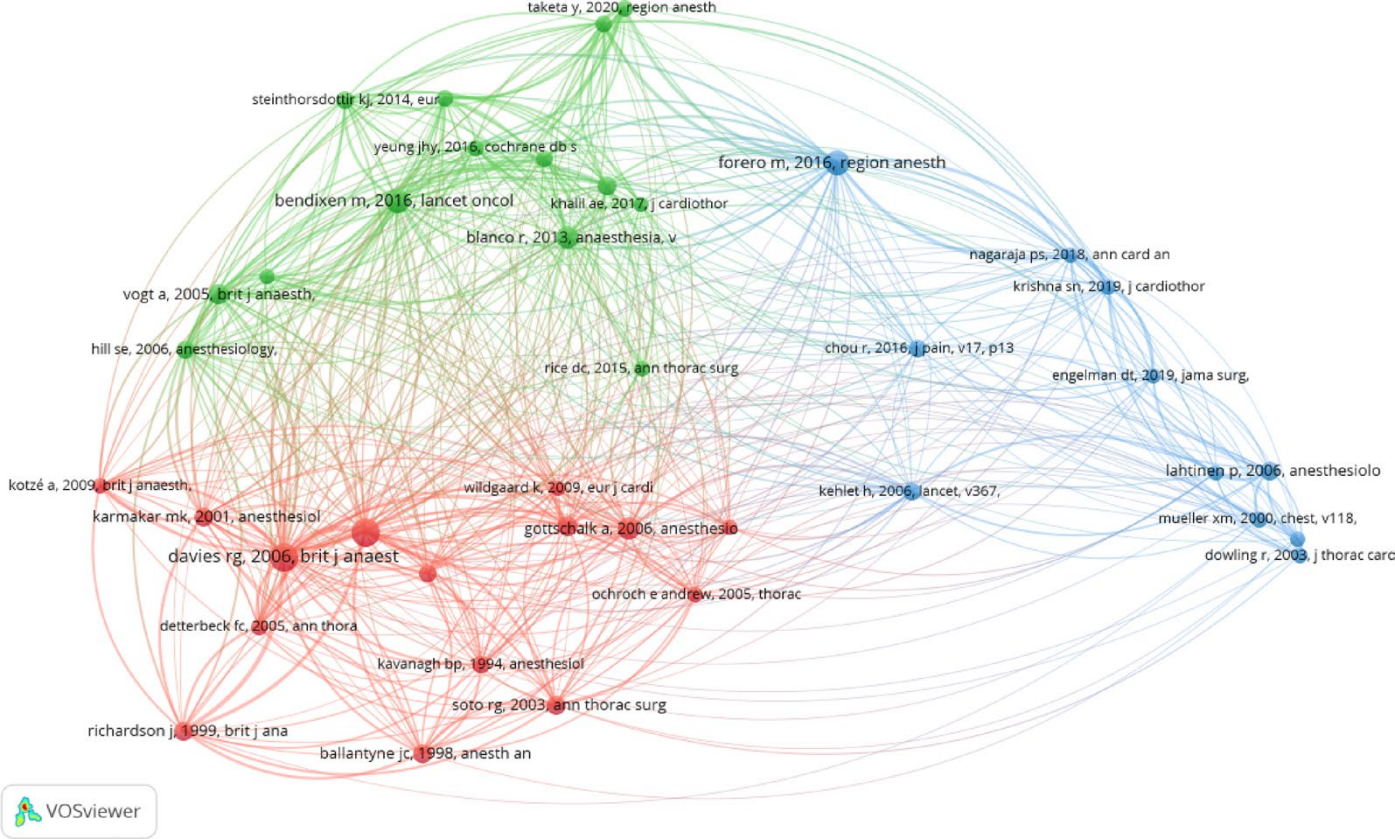
Mapping of co-cited references on acute postoperative pain management following cardiothoracic surgery

We also constructed a visualized map of co-cited references by using VOSviewer . The threshold of the minimum number of co-citations was set to 25. Finally, among the 10,902 cited references, 40 co-cited references were selected for co-citation analysis. The co-citation network map is shown in Fig. 3. The references in the network map were divided into three distinct clusters, which were represented by different colors. The cluster (in red) included 15 references, which mainly concentrated on different nerve block techniques for postoperative analgesia in cardiothoracic surgery. The cluster (in green) addressed the important role of video-assisted thoracoscopic surgery (VATS) in the treatment of lung cancer and in the postoperative pain management. The cluster (in blue) focused on the optimisation of postoperative pain management and its impact on postoperative recovery of cardiothoracic surgery patients.

**Fig. 4:**
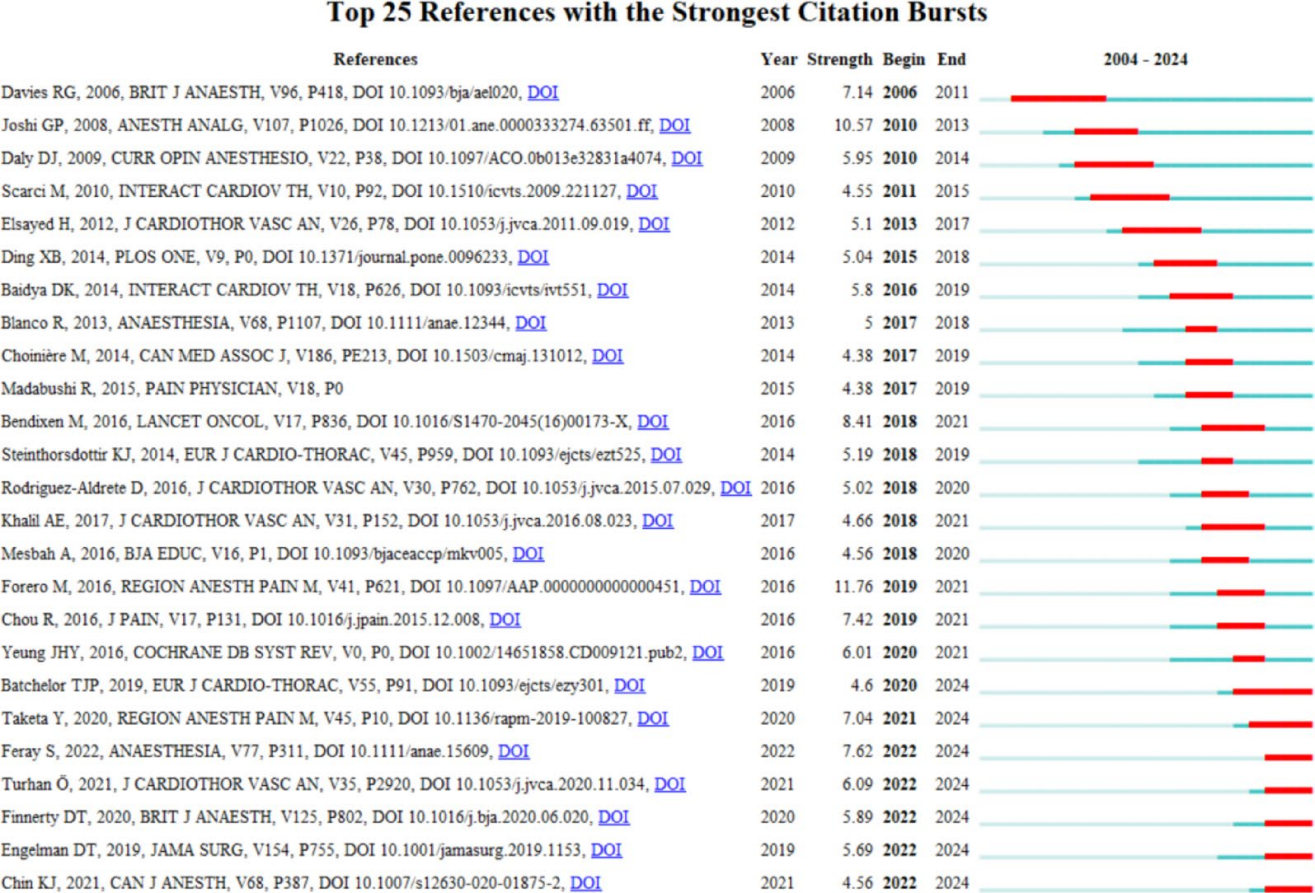
The top 25 references with the strongest citation bursts from 2004 to 2024

Citation burstness refers to references that receive concentrated attention from researchers in a particular discipline over a certain period. In this study, CiteSpace was used to identify references with the strongest citation bursts, with a minimum duration of 1 year for each burst. The top 25 references with the strongest citation were shown in Fig. 4. The reference with the strongest citation strength (strength = 10.57) was “*A Systematic Review of Randomized Trials Evaluating Regional Techniques for Postthoracotomy Analgesia*”, published in *Anesthesia & analgesia* by Joshi et al. in 2008, with a citation burst lasting from 2010 to 2013 [26]. In recent years, the reference with the strongest citation strength (strength = 7.62) was “*PROSPECT guidelines for video-assisted thoracoscopic surgery: a systematic review and procedure-specific postoperative pain management recommendations*”, published in *Anaesthesia* by Feray et al. in 2022, with a citation burst lasting from 2022 to 2024 [27].

### Keyword co-occurrence analysis and keyword burst analysis

**Fig. 5:**
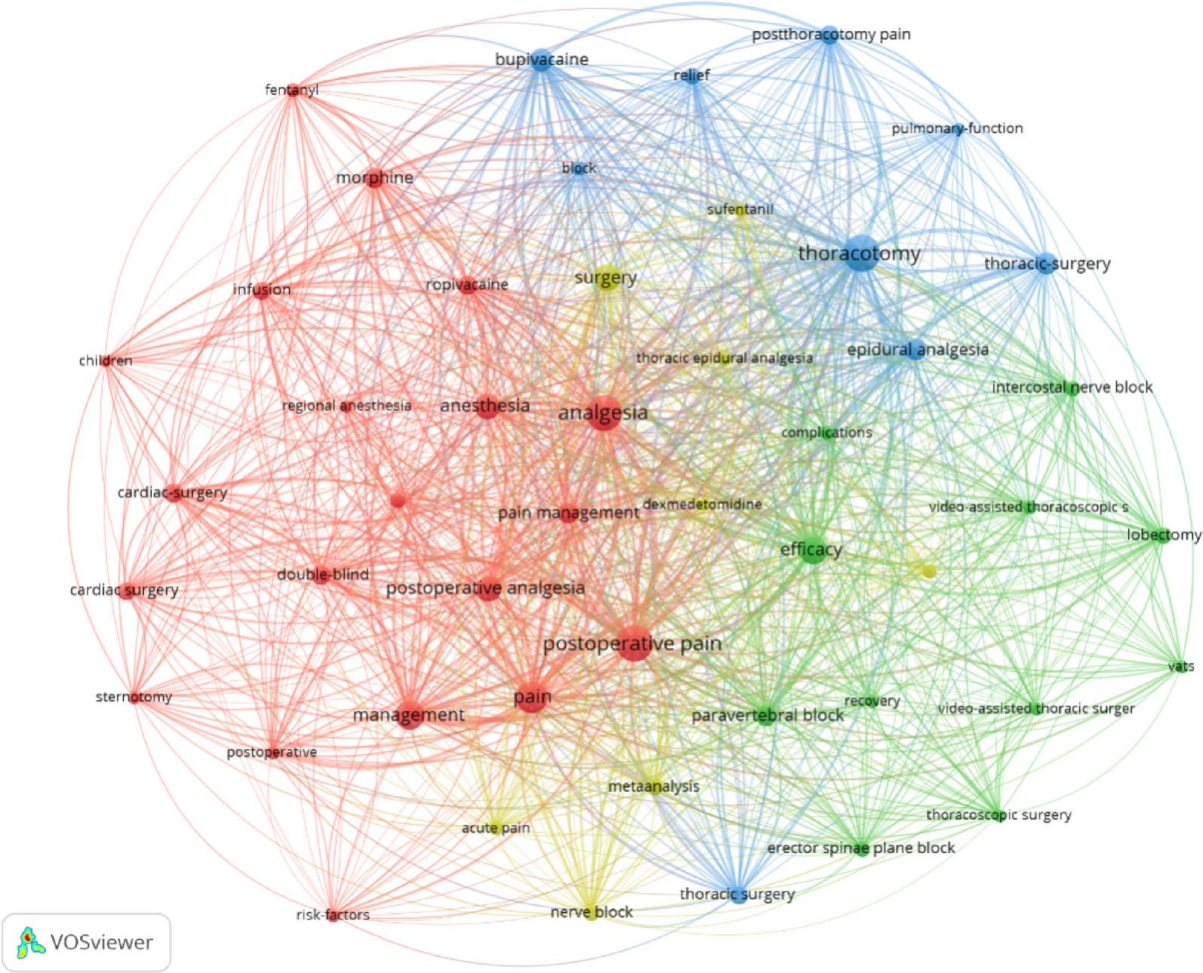
Mapping of keywords on acute postoperative pain management following cardiothoracic surgery

Keywords represent a concise summary of the core information in a publication. And analyzing keywords can reveal research trends and hotspots within a specific scientific field. In this study, VOSviewer was used to perform keyword co-occurrence analysis of the top 48 keywords, selected with a threshold of ≥ 24 occurrences (Fig. 5). In the resulting map, the size of each node represents the frequency of the keyword, with larger nodes indicating more frequently occurring keywords that better reflect research hotspots. The lines between nodes represent the strength of co-occurrence between two keywords, with thicker lines indicating a higher frequency of co-occurrence within the same publication. The color of the nodes represents different clusters, corresponding to distinct research themes. The results show that the top 10 most frequently occurring keywords are *postoperative pain*,* thoracotomy*,* analgesia*,* pain*,* management*,* efficacy*,* surgery*,* anesthesia*,* postoperative analgesia*,* and bupivacaine*. These 48 keywords are divided into four clusters based on their thematic focus: the red cluster focuses on postoperative pain management in cardiac surgery patients, particularly studies on analgesic techniques, drug applications, and efficacy evaluations; the green cluster centers on postoperative pain management and recovery in thoracoscopic surgery; the blue cluster highlights postoperative pain management in thoracic surgery, with a specific focus on pain relief techniques for thoracotomy and their impact on pulmonary function recovery; and the yellow cluster emphasizes acute pain management in thoracic surgery, encompassing analgesic techniques, drug selection, and systematic evaluations of their efficacy.

**Fig. 6:**
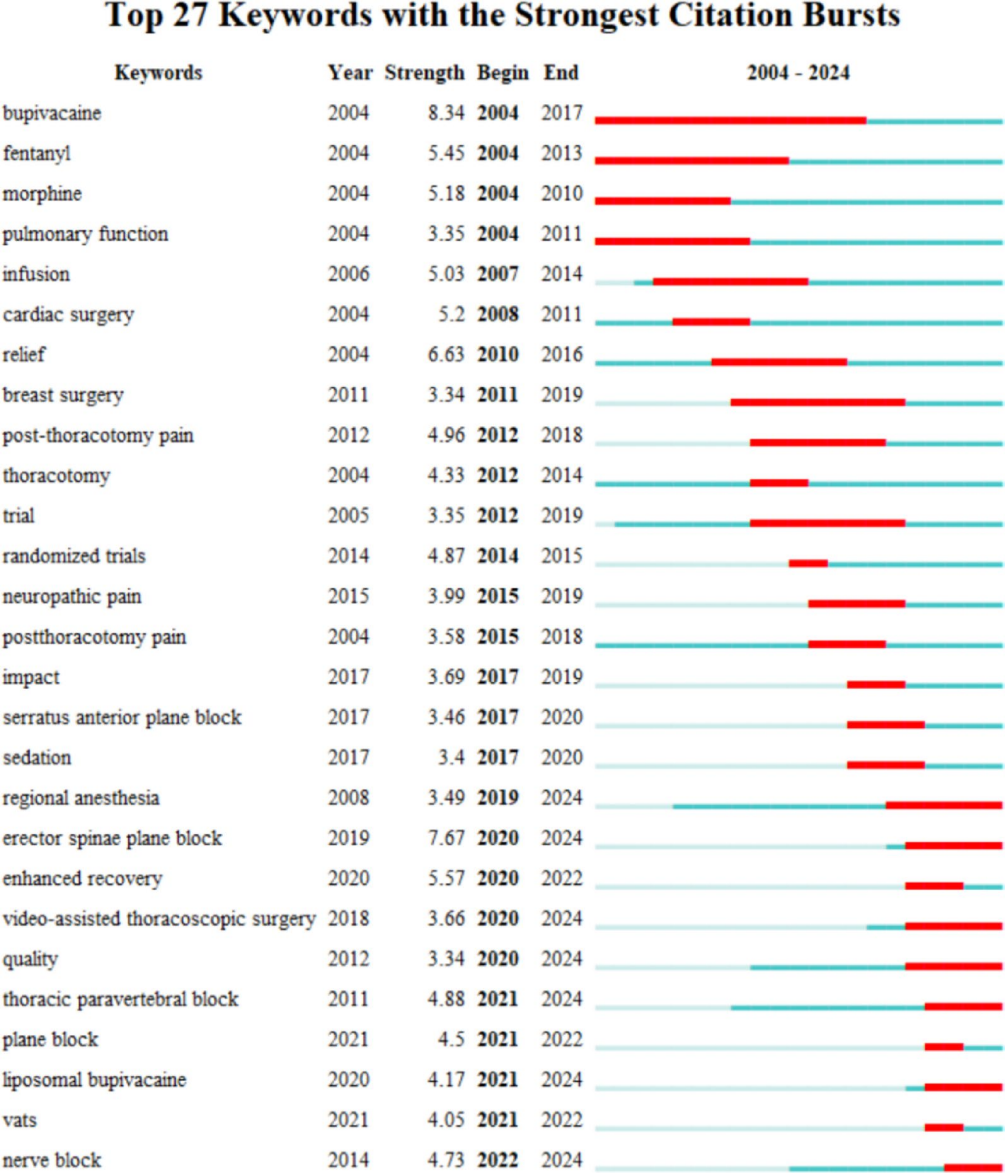
The top 27 keywords with the strongest citation bursts from 2004 to 2024

Keyword burst analysis is carried out to identify keywords that appear with a high frequency within a certain period of time, which helps researchers analyze the evolution of the research. CiteSpace was used for keywords burst analysis. The top 27 keywords that had the strongest citation bursts with a minimum duration of 1 year during the period from 2004 to 2024 were shown in Fig. 6. The top 5 keywords with the highest burst strength were *bupivacaine* (strength, 8.34), *erector spinae plane block* (strength, 7.67), *relief* (strength, 6.63), *enhanced recovery* (strength, 5.57), *and fentanyl* (strength, 5.45). The keywords *bupivacaine* (2004–2017) and *fentanyl* (2004–2013) gained sustained attention in the earlier periods. These drugs were vital for analgesia. In contrast, *erector spinae plane block* (2020–2024), *enhanced recovery* (2020–2024), *thoracic paravertebral block* (2020–2024), *nerve block* (2020–2024), *plane block* (2021–2022), *and liposomal bupivacaine* (2021–2024) were the strongest citation burst keywords in recent years, reflecting emerging trends in acute postoperative pain management after cardiothoracic surgery.

## Discussion

Cardiothoracic surgery can cause moderate to severe pain, and effective postoperative analgesia can improve the clinical prognosis of patients. There has been a significant increase in scientific research on the management of acute postoperative pain after cardiothoracic surgery over the last few years. This study provides a comprehensive overview of acute postoperative pain management following cardiothoracic surgery over the past two decades by using bibliometric analysis.

A total of 740 publications on acute postoperative pain management following cardiothoracic surgery were extracted from the WOS Core Collection database, covering the period from January 1, 2004, to January 1, 2024. Figure 2 shows that the number of publications in this field has grown steadily over year. Between 2004 and 2014, the average annual increase in publications was around 20. After 2015, the number of publications began to rise more sharply, reaching a peak of 96 in 2021. Meanwhile, the total number of citations for annual publications has also risen steadily. This reflects the growing academic value and sustained interest in the topic. Globally, China and the United States are leading countries in this field. They have the highest number of publications and the most active research institutions. Among these institutions, the Cleveland Clinic produced 14 publications, with an average of 29.4 citations per publication, making it one of the most influential institutions in the field. As one of the world’s leading comprehensive medical centers, Cleveland Clinic specializes in the diagnosis and treatment of cardiac diseases as well as in cardiac anesthesia. It has also established one of the largest pain management centers. Its 2020 primary research findings demonstrated that, in minimally invasive thoracic procedures, erector spinae plane block (ESPB) provided better quality of recovery, greater analgesic efficacy, and fewer postoperative complications compared to serratus anterior plane block (SAPB). This study has gained significant influence and been cited 157 times to date [14]. In terms of journals, the *Journal of Cardiothoracic and Vascular Anesthesia* was the most prolific and influential journal in this field. This journal primarily focuses on anesthesia for cardiothoracic surgery and serves as an excellent choice for tracking the latest research progress on acute postoperative pain management following cardiothoracic surgery. Chauhan, S was the most influential author in this field, with the highest number of publications and an average of 70.3 citations per publication. The primary focus of Chauhan, S et al. was on the analgesic efficacy of regional nerve blocks for acute pain following cardiac surgery. In 2019, they conducted the first randomized controlled trial comparing bilateral ESPB with traditional multimodal intravenous analgesia in adult patients undergoing elective cardiac surgery. The results demonstrated that bilateral ESPB effectively reduced acute postoperative pain in this patient population. This study has gained significant attention and has been cited more than 210 times so far [23]. Among the top 10 most-cited references, 7 primarily focus on regional nerve blocks, covering topics such as cardiothoracic epidural analgesia, thoracic paravertebral block (TPVB), SAPB, and ESPB [6, 26, 28–32]. These references emphasise the important role of regional nerve blocks in the field of acute pain management following cardiothoracic surgery. Furthermore, the cluster analysis of co-cited references also highlights that regional nerve blocks represent significant research focus, reflecting their importance in improving postoperative pain control. Historically, thoracic epidural analgesia was widely regarded as the gold standard for postoperative pain relief in cardiothoracic surgery [30, 33]. However, its clinical application has gradually declined due to notable complications, such as hypotension, urinary retention, nausea, and vomiting. As a result, TPVB, with its lower risk of complications, has gradually emerged as a novel alternative for analgesia [28]. A systematic review by Yeung et al. [13] demonstrated that TPVB provided postoperative analgesia comparable to thoracic epidural block for patients undergoing thoracotomy, while significantly reducing the risk of complications such as hypotension and nausea/vomiting. Additionally, the results of a network meta-analysis by Bhushan et al. [34] also revealed that for patients undergoing VATS, TPVB was significantly superior to other regional nerve block techniques in terms of postoperative analgesia. With the increasing popularity of minimally invasive surgical techniques in cardiothoracic surgery [35], such as VATS, novel regional nerve blocks have been investigated. Among these, SAPB, first introduced by Blanco et al. [29], has gained attention as a novel regional block for anterolateral chest wall analgesia. SAPB is characterized by operational simplicity, low complication rate, high safety profile, and significant analgesic efficacy. Additionally, its blockade range encompasses most VATS incisions and chest tube sites, making it a valuable option for acute postoperative pain management in VATS patients [36, 37]. Another novel regional nerve block technique is ESPB, which was first introduced in 2016 [6]. Study by Taketa et al. [38] demonstrated that ESPB provided analgesic effects comparable to TPVB following VATS, with the added advantage of lower plasma concentrations of local anesthetic. Additionally, a systematic review also indicated that ESPB offered satisfactory analgesic effects for patients undergoing cardiothoracic surgery [39].

Through keyword co-occurrence analysis, it was found that the management of acute postoperative pain across different types of surgeries is also a key focus in cardiothoracic surgery analgesia research. Makkad et al. [40] reviewed the latest evidence on acute postoperative pain management in cardiac surgery, highlighting the importance of multimodal analgesia. The authors systematically evaluated various approaches, such as patient-controlled analgesia, regional analgesia, transcutaneous electrical nerve stimulation, music therapy, and massage therapies. Strumia et al. [41]explored safer and more balanced postoperative analgesia strategies for cardiac surgery. Under the multimodal analgesia concept, they advocated combining non-opioid medications (e.g., acetaminophen, nonsteroidal anti-inflammatory drugs, and low-dose ketamine) with regional anesthesia techniques (e.g., fascial plane blocks) to reduce opioid dependence and associated adverse effects. They also recommended integrating these analgesic interventions into Enhanced Recovery After Surgery (ERAS) pathways to accelerate recovery. Feray et al. [27] conducted a systematic review to evaluate the evidence on acute postoperative pain management following VATS and developed optimal practice guidelines. They recommended TPVB or ESPB as the first-line options for postoperative analgesia, intercostal plane block as a secondary option, and opioids for rescue analgesia in emergencies.

Burst analysis of co-cited references and keywords can reveal research frontiers and hotspots in acute postoperative management following cardiothoracic surgery. From 2022 to 2024, the reference with the first-highest burst strength was the article titled *“PROSPECT guidelines for video-assisted thoracoscopic surgery: a systematic review and procedure-specific postoperative pain management recommendations”* [27]. This article recommended multimodal analgesic strategies and individualized pain management approaches, reflecting new trends in pain management after minimally invasive thoracic surgery. The top five keywords with the highest burst strength in recent years were *erector spinae plane block*, *enhanced recovery*, *thoracic paravertebral block*, *nerve block*, and *liposomal bupivacaine*. This indicates that the optimization of regional nerve block techniques, the impact of acute pain management on patients’ recovery quality and quality of life, and the clinical application of novel drugs remain core hotspots for the future.

This bibliometric analysis indicates a rapid increase in publications on ERAS in the field of cardiothoracic analgesia, which suggests that the management of acute postoperative pain following cardiothoracic surgery is transitioning from the traditional opioid analgesia to multimodal analgesia. As a multimodal perioperative medical enhancement program, ERAS primarily integrates multimodal analgesia and regional nerve block techniques in cardiothoracic analgesia [42, 43]. It aims to reduce perioperative postoperative pain and complications, thereby accelerating patient recovery. Current research indicates that implementing ERAS after thoracic surgery improves pain control and reduces postoperative opioid consumption [10]. Although numerous studies have examined individual components of ERAS in cardiothoracic analgesia in recent years, some findings remain inconclusive or conflicting. Future research should not only comprehensively evaluate the clinical benefit of each ERAS intervention and integrate ERAS pathway for specific surgeries, but also design well-conducted clinical trials to further validate the overall benefits of the EARS programs. Concurrently, risk-stratification tools are essential to guide individualized ERAS implementation for high-risk versus low-risk patients.

The results of this bibliometric analysis also indicate a rapid increase in publications on ESPB, which demonstrates that ESPB could be a promising alternative to epidural analgesia or TPVB for pain management in cardiothoracic surgery. However, most of the current studies are small sample-size trials or reviews [44–46]; there is a lack of large-scale comparative trials between ESPB and TPVB, and data on the effects of ESPB on postoperative complications, quality of life, and chronic postoperative pain are insufficient. Consequently, there is no high-quality research available to assess its clinical benefits. Future research should integrate imaging techniques to elaborate the unique anatomical courses and distributions of the erector spinae muscle, its fascia, and related nerves. Multicenter clinical studies are needed to obtain high-quality evidence guiding clinical application.

As one of the emerging research hotspots, the number of publications on liposomal bupivacaine (LB) is on the rise. As a novel long-acting local anesthetic, a clinical study by Chi Y et al. has demonstrated that LB can prolong postoperative analgesia duration (up to 72 h) and reduce early postoperative opioid consumption, thereby addressing the shortcomings of conventional local anesthetics with limited duration of action [47]. Although it has been applied in cardiothoracic surgery, existing clinical studies remain inconclusive regarding its sustained analgesic efficacy. Some meta-analyses and trials have found that LB is not superior to conventional local anesthetics or alternative analgesic regimens for postoperative analgesia and other secondary clinical outcomes [48–50]. The reasons for these efficacy differences may be related to heterogeneity in administration routes (block site, dosage, and timing) or patient factors. Future studies should further evaluate the pharmacokinetics and safety of LB in special populations (pediatric or elderly patients), optimize administration routes, and explore their effects on chronic pain.

There are still several key limitations in the present study. First, our study was restricted to literature only from the WOS Core Collection, which may induce selection bias. In future work, we plan to include more databases (such as MEDLINE) to provide a more comprehensive analysis. Second, we limited our analysis to studies written in English. As a result, research published in other languages may have been excluded, which could induce bias. Third, our analysis is subject to temporal bias: newer studies have had less time to accumulate citations, and the database is updated dynamically, thus some recently published or subsequently highly cited studies may have been omitted from our analysis at the time of our search. Consequently, observed citation bursts may be amplified or attenuated as databases are updated. we recommend cautious interpretation of citation bursts. Fourth, bibliometric citation counts do not directly reflect the quality or clinical impact of a publication.

## Conclusions

This study analyzed the literature on acute postoperative pain management following cardiothoracic surgery and revealed a significant increase in attention to this field in recent years. The United States and China were the leading countries in terms of productivity and influence. Future research directions focus on enhanced recovery, multimodal analgesia, regional nerve block, and the development of novel local anesthetics, which warrant further in-depth exploration.

## Supplementary Information

Supplementary Material 1

## Data Availability

The raw data underlying this study were licensed from the Web of Science for academic use only and are not publicly available due to these licensing restrictions. However, the data that support the findings are available from the corresponding author upon reasonable request.
