# Supplementary material for: Acute postoperative pain management after cardiothoracic surgery: a bibliometric analysis and future directions
Source: J Cardiothorac Surg. 2025 Nov 27;20:477. doi: 10.1186/s13019-025-03734-x (PMC12750945; doi:10.1186/s13019-025-03734-x)
Supplement: Supplementary file 1 — Supplementary Material 1 [file 13019_2025_3734_MOESM1_ESM.docx]

| Table S1. Search strategies for bibliometric analysis. | |
| --- | --- |
| Database: | Web of Science Core Collection |
| Retrieval mode: | Advanced Search |
| Retrieval strategy: | #1:TS=(“pain” OR “postoperative pain” OR “acute postoperative pain” OR “analgesia” OR “analgesic” OR “pain management” OR “pain control” OR “patient-controlled analgesia” OR “regional analgesia” OR “neuraxial analgesia” OR “thoracic epidural analgesia” OR “intercostal analgesia” OR “intercostal nerve block” OR “thoracic paravertebral nerve block” OR “serratus anterior plane block” OR “erector spinae plane block” OR “multimodal analgesia” )  #2:TS=(“postoperative” OR “postoperative periods” )  #3:TS=(“Thoracic Surgery” OR “thoracoscopy” OR “thoracotomy” OR “Video-thoracoscopic Surgery” OR “Video-Assisted Thoracoscopic Surgery” OR“VATS” OR “Video-assisted thoracic surgery” OR “Anatomic lung resection” OR “lobectomy” OR “segmentectomy” OR “lung resection” OR “pulmonary surgical procedures” OR “cardiac surgical procedures” OR “cardiac surgery” OR “esophagectomy” OR “mediastinoscopy” OR “sternotomy” OR “thoracoplasty” OR “thoracostomy” OR “thoracotomy” OR “thymectomy”)  #4: LA = (English)  #5: DT = (Article)  #6:PY= (2004-2024)  Final = #1 AND #2 AND #3 AND #4 AND #5 AND#6: |
